# Senning Procedure: Conceptualization in the Wet Lab

**DOI:** 10.21470/1678-9741-2023-0025

**Published:** 2023-10-02

**Authors:** César Castillo Romero, Iris Pamela Flores Sarria, Gabriella Ricciardi, Jorge Luis Cervantes Salazar

**Affiliations:** 1 Department of Congenital Heart Surgery, National Institute of Cardiology Ignacio Chávez, Mexico City, Mexico; 2 Department of Cardiac Surgery, University of Lille, Lille University Hospital, Lille, France

**Keywords:** Animal Model, Congenital Heart Disease, Pediatric

## Abstract

Training congenital heart surgeons today is challenging for themselves and their mentors. The situation becomes even more complicated while teaching complex surgical procedures. Senning operation is one of the most ingenious intracardiac techniques. We consider this surgical technique a worthy example to stand out the potential advantage of wet lab training. This article demonstrates the simulation of the Senning procedure in an explanted porcine model.

## INTRODUCTION

**Table:** 

Abbreviations, Acronyms & Symbols	
CS	= Coronary sinus
CT	= Crista terminalis
IAS	= Interatrial septum
IVC	= Inferior vena cava
LA	= Left atrium
LIPV	= Left inferior pulmonary vein
lp	= Lower part
LSPV	= Left superior pulmonary vein
RA	= Right atrium
RAAW	= Right atrial anterior wall
RALW	= Right atrial lateral wall
RIPV	= Right inferior pulmonary vein
RPV	= Right pulmonary vein
RSPV	= Right superior pulmonary vein
SVC	= Superior vena cava
tp	= Top part

The education of young congenital heart surgeons under current residency programs does not allow them to gain the necessary experience to perform complex procedures during their training^[1]^. Senning operation is one of the most ingenious intracardiac techniques. The atrial switch procedure was once frequently performed by almost all cardiac centers but recently it has become an uncommon surgery carried only in specialized centers undertaking repair of congenitally corrected transposition of the great arteries. Due to limited exposure and technical complexity, the Senning operation is considered a challenging procedure for young surgeons^[2]^. We consider this surgical technique a worthy example of the importance of deliberate practice in the wet lab^[3^,^4]^. This article demonstrates the feasibility of the Senning procedure in the porcine model and that it can be a useful tool to conceptualize the surgical technique. This report was approved by the local institutional review board. The approval included a waiver of informed consent because it does not show personal data of any patient.

## TECHNIQUE

An explanted postmortem porcine heart (from a local butchery) was used. Despite the presence of normally related great arteries, the surgical principle of the Senning procedure is adequately simulated:

1. The Waterston’s groove is dissected as deep as possible without entering the left atrium (LA). The right atrium (RA) is open anteriorly (5 to 10 mm) and parallel to the crista terminalis (Figure 1A and Video 1). The distance between the atrioventricular groove and the first incision should correspond to approximately two-thirds of the circumference of the superior vena cava (SVC). The atrial septal flap (first flap) is developed (Video 2): fossa ovalis and the coronary sinus (CS) should be identified (Figure 1B). Superiorly, the incision of the limbic tissue must be done toward and within the superior vena caval orifice, inferiorly the incision should be extended to the junction between the inferior vena cava (IVC) and the right inferior pulmonary vein (RIPV).
  **Fig. 1:**
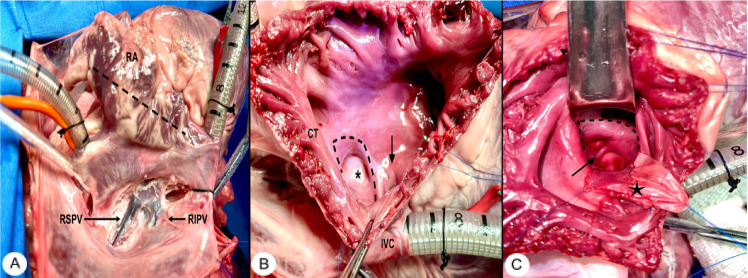
(A) Dissection of interatrial groove and right atrial initial incision. Dotted lines indicate the site of the first incision. (B) Localization of limbic tissue. The asterisk indicates fossa ovalis; the dotted lines, incision to create the atrial septal flap; and the arrow, coronary sinus. (C) Creation of atrial septal flap. Dotted lines indicate the site where the flap will be sutured; the arrow, left pulmonary veins; and the star, the flap extended with a bovine pericardium patch. CT=crista terminalis; IVC=inferior vena cava; RA=right atrium; RIPV=right inferior pulmonary vein; RSPV=right superior pulmonary vein.
  **Video 1:**
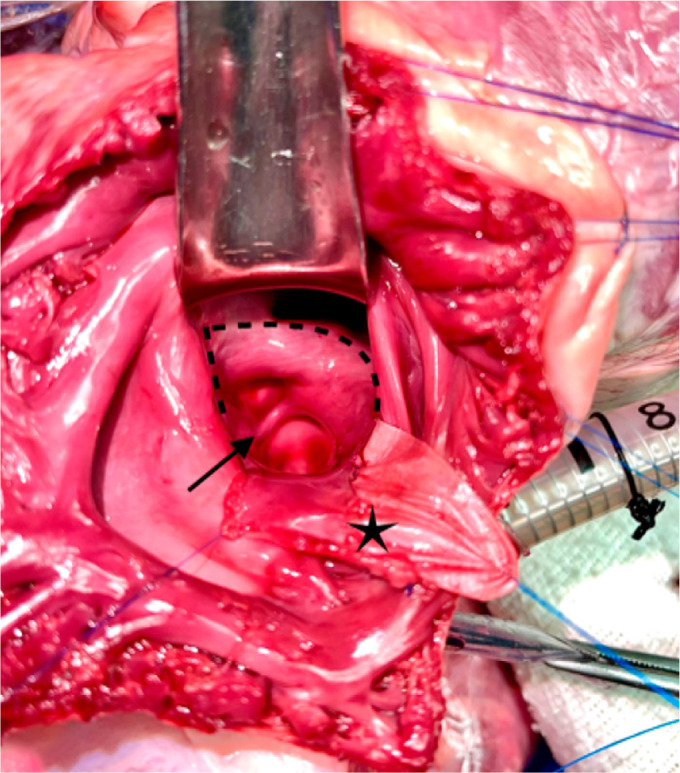
This clip shows the procedure’s initial steps: dissection of Waterston’s groove, right atriotomy, and preparation for the first flap. Link: https://s3.sa-east-1.amazonaws.com/publisher.gn1.com.br/bjcvs.org/videos/e20230025VideoPart1.mp4
  **Video 2:**
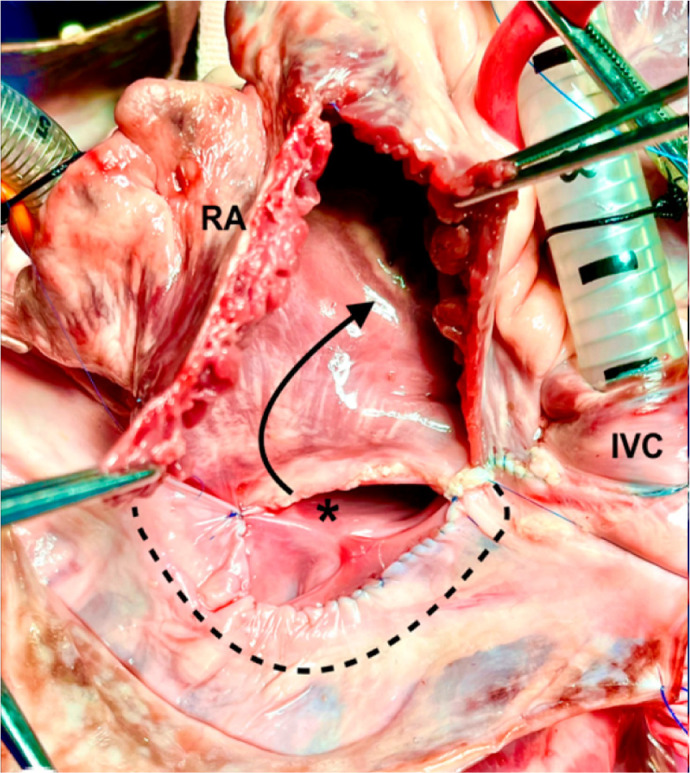
Creation of atrial septal flap. The flap was sutured between the left pulmonary veins and the left atrial appendage. Link: https://s3.sa-east-1.amazonaws.com/publisher.gn1.com.br/bjcvs.org/videos/e20230025VideoPart2.mp4
2. The atrial flap is extended with a small triangular patch of bovine pericardium (Figure 1C).
3. The atrial septal flap is sutured between the left pulmonary veins and the left atrial appendage. Superiorly, the flap is sutured to the junction between the SVC and right superior pulmonary vein, inferiorly to the intersection between the IVC and the RIPV (Figure 2A and Video 2).
  **Fig. 2:**
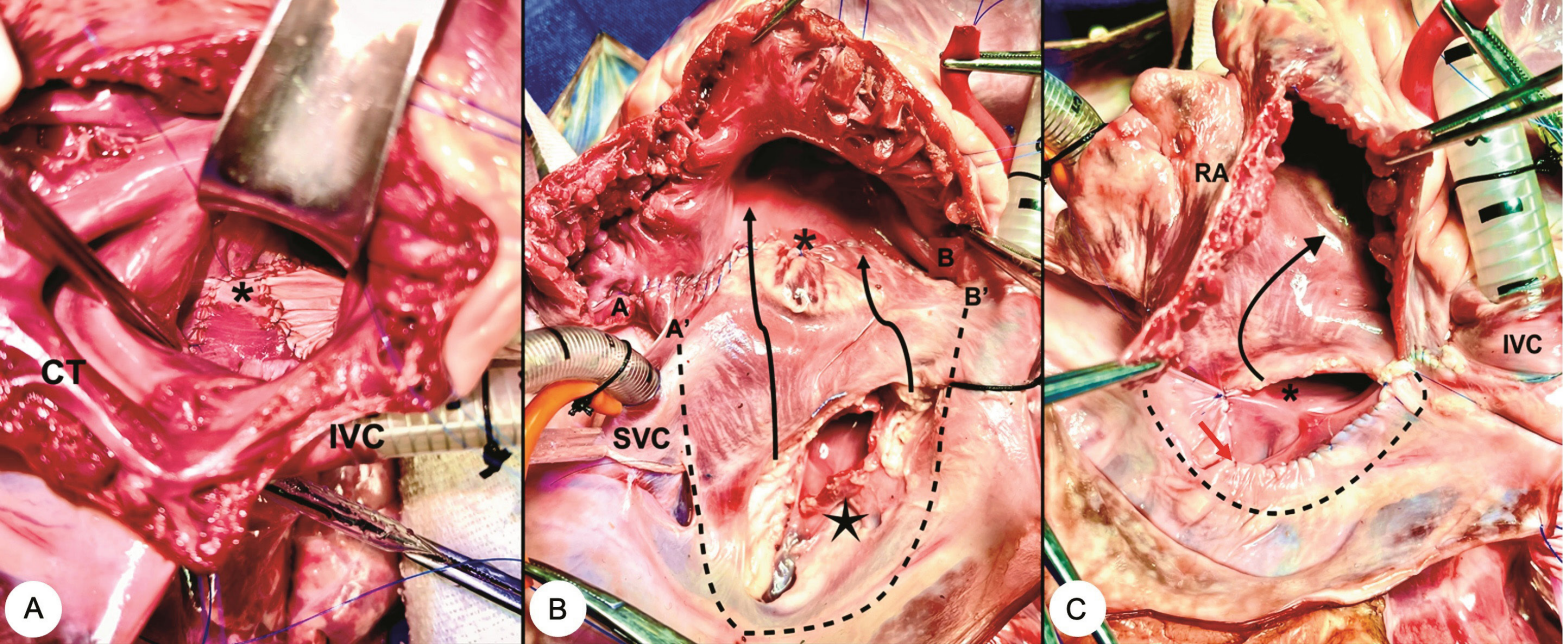
(A) Atrial septal flap sutured. Asterisk indicates the flap sutured between the left pulmonary veins and the left atrial appendage. (B) Enlargement of the left atrium and systemic venous pathway completed. The asterisk indicates second flap suture; the star, left atrium and right pulmonary veins enlarged; arrows, the new pulmonary venous course; points A and B´, augmentation incisions and suture sites for each increased flap extremity; and dotted lines, the area where the anterior wall of the right atrium will be sutured. (C) Reconstruction of pulmonary venous atrium. The red arrow indicates the coping of edges of the right pulmonary veins to the pericardial reflection; the asterisk, left atrium; dotted lines, the suture line site for the third flap; and the black arrow, the new pulmonary venous pathway. CT=crista terminalis; IVC=inferior vena cava; RA=right atrium; SVC=superior vena cava.
4. The LA is enlarged by incising both right pulmonary veins (RPVs).
5. The right atrial lateral wall (second flap) is sutured along with the anterior remnant of the limbic tissue, staying away from the atrioventricular node (systemic venous pathway finished). The CS is left opening into the new pulmonary venous atrium (Figure 2B).
6. Reconstruction of the pulmonary venous atrium: the right atrial incision is extended at its ends by two anterior incisions to augment the perimeter of the atrial flap (points A and B in Figure 2B). Inferiorly, the flap is sewn on the lateral aspect of the IVC, superiorly the suture line is put on the SVC in front of the cavoatrial junction. Posteriorly, pericardial reflection is used as an extension: it is sewn to the edges of RPVs and LA to obtain as large an opening as possible (Figure 2C).
7. The right atrial anterior wall (third flap) is sutured to the pericardium. The pulmonary venous pathway has now been completed (Figure 3A and Video 3). Figure 3B illustrates the new systemic and pulmonary pathways created by the tunnels in the Senning procedure.
  **Video 3:**
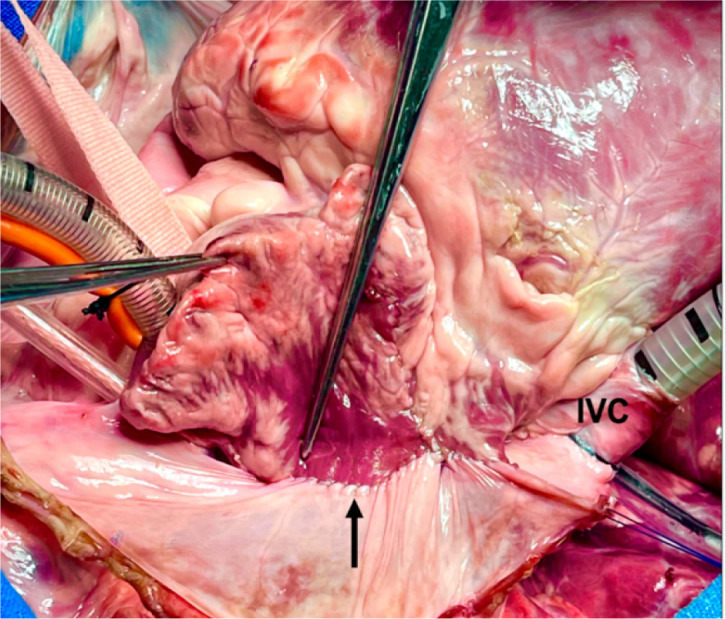
This clip shows the enlargement of the left atrium and a new systemic venous pathway, the reconstruction pulmonary venous atrium, the coping of edges of the right pulmonary veins to the pericardial reflection, and the final aspect after the procedure. Link: https://s3.sa-east-1.amazonaws.com/publisher.gn1.com.br/bjcvs.org/videos/e20230025VideoPart3.mp4
  **Fig. 3:**
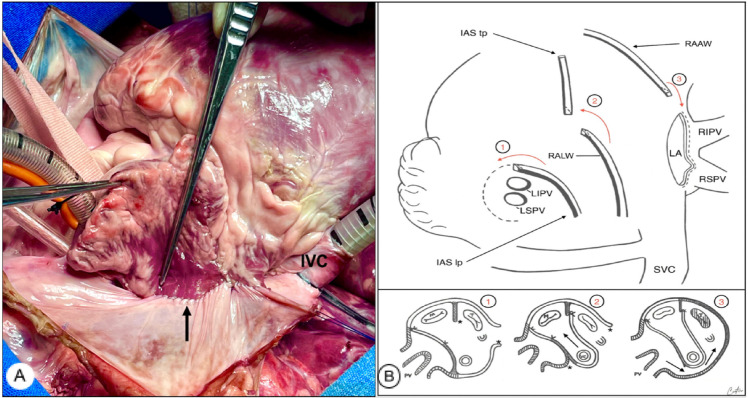
(A) Final aspect after the procedure. The arrow indicates the suture line of the last flap. (B) Schematic drawings of the Senning procedure. Upper panel shows the shift (red arrows 1, 2, and 3) of flaps toward the structures in which they will suture them. In the lower panel, the cross-sectional view shows the intracardiac tunnels and systemic and pulmonary pathways (black arrows). IAS=interatrial septum; IVC=inferior vena cava; LA=left atrium; LIPV=left inferior pulmonary vein; lp=lower part; LSPV=left superior pulmonary vein; RAAW=right atrial anterior wall; RALW=right atrial lateral wall; RIPV=right inferior pulmonary vein; RSPV=right superior pulmonary vein; SVC=superior vena cava; tp=top part.

## DISCUSSION

Congenital heart surgery is technically demanding. The most significant benefit of deliberate practice arises precisely for highly complex procedures^[4,5]^. The preparation of young surgeons with the current residency programs does not allow them to gain a large experience to perform complex operations such as the Senning procedure, due to the limitation of the surgical exposure in the operating room. However, the naive young surgeon could be in the challenging setting of performing this operation sooner or later^[1]^. The neuropsychological concept emanating from *deliberate practice* has been widely investigated. It does not represent a simple training activity; the process comprises mental representations of the procedure, planning the action, executing the operation, analyzing the mistakes, and carrying out the process again^[4]^. These learning steps need to be always supervised by an expert senior surgeon^[5]^. The atrial switch simulation is an exceptional example of the importance of wet lab training because it is possible not only to understand a complex procedure but also to practice it reliably. One can even simulate modifications as we did with the extension of the pericardial reflection. In addition, the young surgeon can learn the critical steps of the procedure, such as the site, shape, and length of the first incision, because an inappropriate cut in the RA (first incision) could result in a lateral portion of the RA insufficient to perform the last flap. Then there is a need to use prosthetic material. We consider that the characteristics of the porcine RA, rather than a limitation, it’s a good anatomical model for training this crucial step. It is a priority to have a model with preserved lung block and pericardium because this will allow fixation of the pericardium for better surgical exposure and a higher level of simulation. In addition, the pericardium will be available if required at the end of the procedure. Preservation of the pulmonary block is essential to maintain the anatomy of the pulmonary veins and enable the procedure to be carried out. Like any complex process, the Senning procedure must be perfectly conceptualized before its execution, and training in the wet lab is a valuable tool for this purpose. We trust that our images, diagram, and video will be constructive in facilitating your understanding.

## CONCLUSION

The simulation of the Senning procedure in a porcine model allows training in the wet lab and assistances to understand the surgical principle of this complex procedure. It is our belief this learning method can be replicated without trouble.

## Abbreviations, Acronyms & Symbols

CS: = Coronary sinus
CT: = Crista terminalis
IAS: = Interatrial septum
IVC: = Inferior vena cava
LA: = Left atrium
LIPV: = Left inferior pulmonary vein
lp: = Lower part
LSPV: = Left superior pulmonary vein
RA: = Right atrium
RAAW: = Right atrial anterior wall
RALW: = Right atrial lateral wall
RIPV: = Right inferior pulmonary vein
RPV: = Right pulmonary vein
RSPV: = Right superior pulmonary vein
SVC: = Superior vena cava
tp: = Top part
